# Short term but highly efficient Cas9 expression mediated by excisional system using adenovirus vector and Cre

**DOI:** 10.1038/s41598-021-03803-w

**Published:** 2021-12-21

**Authors:** Sayaka Nagamoto, Miyuki Agawa, Emi Tsuchitani, Kazunori Akimoto, Saki Kondo Matsushima, Yumi Kanegae

**Affiliations:** 1 Core Research Facilities, Research Center for Medical Sciences, The Jikei University School of Medicine, Tokyo, Japan; 2 Faculty of Pharmaceutical Sciences, Tokyo University of Science, Chiba, Japan; 3 Division of Gene Therapy, Research Center for Medical Sciences, The Jikei University School of Medicine, Tokyo, Japan

**Keywords:** Genetic engineering, Genetic techniques, Microbiology techniques

## Abstract

Genome editing techniques such as CRISPR/Cas9 have both become common gene engineering technologies and have been applied to gene therapy. However, the problems of increasing the efficiency of genome editing and reducing off-target effects that induce double-stranded breaks at unexpected sites in the genome remain. In this study, we developed a novel Cas9 transduction system, Exci-Cas9, using an adenovirus vector (AdV). Cas9 was expressed on a circular molecule excised by the site-specific recombinase Cre and succeeded in shortening the expression period compared to AdV, which expresses the gene of interest for at least 6 months. As an example, we chose hepatitis B, which currently has more than 200 million carriers in the world and frequently progresses to liver cirrhosis or hepatocellular carcinoma. The efficiencies of hepatitis B virus genome disruption by Exci-Cas9 and Cas9 expression by AdV directly (Avec) were the same, about 80–90%. Furthermore, Exci-Cas9 enabled cell- or tissue-specific genome editing by expressing Cre from a cell- or tissue-specific promoter. We believe that Exci-Cas9 developed in this study is useful not only for resolving the persistent expression of Cas9, which has been a problem in genome editing, but also for eliminating long-term DNA viruses such as human papilloma virus.

## Introduction

The usefulness of genome editing technology has been manifest not only in basic science but also in gene therapy^1,2^. The CRISPR/Cas9 system in particular has become widespread because of its ease of use. CRISPR/Cas9, which is composed of the Cas9 nuclease and a guide RNA, was originally discovered as part of an adaptive immune system of bacteria and archaea against viruses and plasmids^3,4^. When the two factors, Cas9 nuclease and a guide RNA against a target DNA, are introduced into cells, site-specific double-stranded breaks (DSB) in the genome can be induced efficiently^5–8^. When DSB is induced, the genome is repaired by the non-homologous end-joining (NHEJ) and homology-directed repair (HDR) pathways. As a DNA repair mechanism, the NHEJ pathway is very efficient, but often results in random small insertions or deletions (indels). Since indels in exons can induce frameshift mutations, genome editing is widely used to easily make knockout cells or knockout mice^9,10^. In addition, the repair mechanism via HDR is also used for knock-in technology^11,12^.

An efficient Cas9 transduction system is still a remaining problem in genome editing. Although plasmid transfection or electroporation are widely used in vitro, high editing efficiency is not achievable in cells that show low transfection efficiency, and its application in vivo is difficult except for fertilized eggs. For in vivo applications, virus vectors have been applied. Since the DNA size of the commonly used SpCas9 derived from *Streptococcus pyogenes* is about 4.3 kb, which exceeds the insertion DNA size of adeno-associated virus (AAV) vectors, smaller sized Cas9 DNAs like SaCas9 derived from *Staphylococcus aureus* are used^13,14^. For the expression of SpCas9, the lentiviral vector has been applied^15,16^. However, both AAV and lentiviral vectors are long-term expression vectors, so there are concerns when applying them to genome editing. The continuous expression of Cas9, which has DSB activity, may increase the risk of off-target effects^17,18^. Therefore, the development of highly efficient and short-term Cas9 expression vector systems is needed.

However, there have been fewer reports of adenovirus vector (AdV) use in genome editing compared to other vectors^19–23^. Although AdV has been reported to induce strong inflammation by AdV itself, this inflammation is easily reduced by changing the promoter used for gene expression. Nakai et al*.* reported that the EF1α promoter in AdV does not show strong inflammation while increasing ALT expression in vivo. Although AdV is a transient expression vector, the period of gene expression persists for about 6 months when the EF1α promoter is used^24^. If AdV and a short-term expression system can be combined, the usefulness and safety of genome editing may be enhanced.

In this study, we constructed a novel Cas9 expression system combining AdV and the site-specific recombinase Cre. This system showed short-term and strong expression of Cas9. To examine the usefulness of this system, we applied it to the cleavage of the hepatitis B virus (HBV) genome, which causes viral hepatitis B. Hepatitis B currently has more than 200 million carriers in the world and can progress to liver cirrhosis or hepatocellular carcinoma with high probability^25–27^. Because AdV shows highly efficient gene transduction in liver-derived cells, the cleavage of the HBV genome can verify the efficacy of this system. In the treatment of hepatitis B, it is extremely difficult to eliminate cccDNA, which are HBV genomes that remains extra-chromosomally in hepatocyte cell nuclei over the long term and continue to supply viral genomes^28^. Genome editing may be very useful for the complete elimination of cccDNA in hepatocytes^29–35^.

The system developed here, which expresses Cas9 for a short time, succeeded not only in efficiently removing replicating HBV genomes, but also in hepatocyte-specific genome editing.

## Results

### Induction of short-term but highly efficient genome editing by “Excisional” expression

The efficiency of genome editing using AdV was compared with that of commonly used plasmid transfection. After GFP-expressing AdV was used to infect Huh-7 cells, we confirmed by GFP expression that AdV was introduced into all cells. As the Cas9-plasmid, Cas9/gRNA-G-1, which has a Cas9 expression unit and a G1 guide RNA against GFP (Supplementary Table S1) on the same molecule, was used to transfect cells. As the Cas9-AdV, Avec-Cas9, which expresses SpCas9 driven by the CBh promoter, was used to coinfect cells with gRNAG1-Cre AdV, which carries the same guide RNA as the plasmid (Supplementary Fig. S1). For the plasmid, cells transfected with 0.3 μg DNA showed the highest indel introduction efficiency at 9.3%. In contrast, for AdV, cells infected at MOI 0.3 already showed 9.7% indel introduction. These results confirmed that AdV is an excellent tool that can increase the efficiency of genome editing.

**Figure 1:**
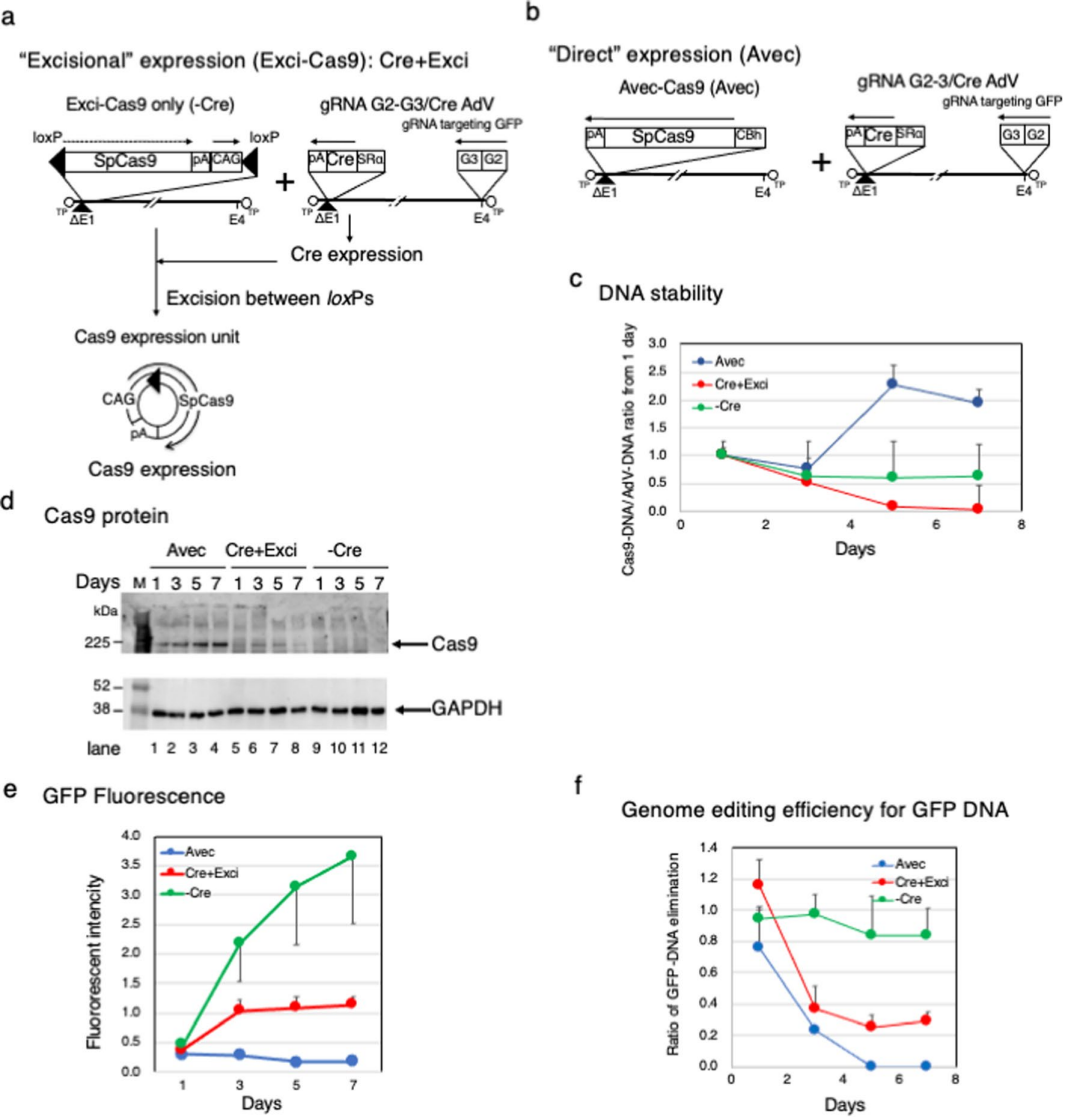
Short-term Cas9 expression and highly efficient genome editing using “Excisional” expression. (**a**) Schema of the “Excisional” Cas9 expression system. (**b**) Schema of “Direct” expression for Cas9. (**c**) DNA stability of the introduced Cas9 expression gene. The blue line is Cas9 DNA derived from Avec. The red line is a circular molecule with the Cas9 expression unit excised by Cre. The green line is Cas9 DNA from AdV without Cre introduced. (**d**) Time course of expressed Cas9 protein. GAPDH was used as an endogenous control (Full-length blot was showed as Supplementary Fig. S4). (**e**) Changes in GFP fluorescence by genome editing. (**f**) Remaining amounts of GFP DNA after genome editing.

Although AdV is classified as a transient gene expression vector, AdV genomes are relatively stable in cells compared to plasmid DNA^36^. Therefore, we applied an “Excisional” expression system that we developed and reported before^37^ and constructed an “Excisional Cas9” system, named Exci-Cas9 that expresses Cas9 protein strongly over a short period. This system by itself did not express Cas9 (Fig. 1a, left), because Cas9 cDNA was in front of the CAG promoter. However, when Cre was supplied from gRNA/Cre AdV (Fig. 1a, right), strong expression of Cas9 was induced, because Cas9 and the CAG promoter were joined in the normal order. So, in this system, the Cas9 expression unit was present in the circular molecule excised by Cre (Fig. 1a, bottom). In other words, this method increases the transfection efficiency of the plasmid, and it can be expected that the Cas9 expression period will be shortened.

Therefore, we compared the expression period of Cas9 between a “Direct” expression system (Avec) (Fig. 1b), in which Cas9 is expressed directly from the CBh promoter and “Excisional” expression system (Cre + Exci) (Fig. 1a). In this experiment, we used two gRNAs named G2 and G3 (Supplementary Table S1) arranged in tandem. One day after infecting Huh-7 cells with GFP-AdV, gRNA-G2-3/Cre AdV (Cre) and Avec-Cas9 (Avec), or Cre and Exci-Cas9 (Cre + Exci) were each used for coinfection at MOI 7. -Cre was a control in which Cas9 expression units were not generated because Cre was not transduced, and Exci-Cas9 and control AdV (Ad1w1), which did not have an inserted gene, were used for coinfection. Infected cells were harvested 1, 3, 5, and 7 days after Cas9 transduction.

In this study, we used multiplex gRNAs to increase the efficiency of genome editing. When the indel (%) was quantitated by T7E1 assay, induced indels by AdV carrying a single gRNA were 11.5% for G2 and 23.5% for G3. However, with AdV that has multiplex gRNA G2-G3 (G2 and G3 on the same molecule), the indel percent was 57.3%, which was about twice that of a single gRNA (Supplementary Fig. S2).

The amount of remaining DNA of Cas9 was calculated using a primer for Cas9 and a primer for the AdV backbone. In Fig. 1c, the amount of Cas9 DNA on each day is shown as the value when the ratio of the number of copies of Cas9 and AdV on day one was set as 1. That means that the value was the copy number of Cas9 DNA compared to that of AdV DNA on each day, and as a result, the decrease in AdV DNA due to cell division for example was corrected. In all groups, the amount of Cas9 DNA was similar to that of AdV from 1 to 3 days after transduction. The Cas9 DNA of Cre + Exci decreased from 5 days and was hardly detected at 7 days after infection. In contrast, Cas9 DNA of -Cre during that period was mostly stable. Avec showed a two-fold increase in Cas9 DNA, suggesting that Cas9 mRNA might have remained after RNase treatment during DNA extraction.

Cas9 protein in Avec was detected from day 1 and was stably detected until 7 days later (Fig. 1d, lanes 1–4). In -Cre, Cas9 was not detected at any time point (Fig. 1d, lanes 9–12). This result showed that there was no Cas9 leakage expression from the “Excisional” system unless Cre was introduced. While the Cas9 expression of Cre + Exci, which was coinfected with gRNA and Cre, was not detected on day 1, Cas9 protein started to be detected at 3 days then decreased continually at 5 and 7 days (Fig. 1d, lanes 5–8). From these results, we consider Exci-Cas9 to be a highly useful system for expressing Cas9 strongly in a short period as compared with Avec.

The efficiency of genome editing against GFP was compared between Avec-Cas9 and Exci-Cas9 (Fig. 1e). GFP expression increased over time in -Cre, because it did not express Cas9. In Avec, GFP expression was not found. In contrast, in Cre + Exci, GFP expression increased until day 3, consistent with the result of western blotting, but no increase was observed thereafter.

The amount of GFP-DNA remaining without genome editing was quantified using a primer set designed for the DNA region between guide RNA G2 and guide RNA G3 (Fig. 1f) (Supplementary Table S2). As with GFP expression, the amount of GFP-DNA remaining after genome editing decreased to 0.76 on day 1 in Avec and was not detected after day 5. In Cre + Exci, GFP-DNA remained stable after day 1 and rapidly decreased to 0.37 after day 3. However, GFP-DNA remained stable in -Cre. These results show that Exci-Cas9 is a short-term high expression system of Cas9 controlled with high accuracy by Cre and with high efficiency of genome editing.

### Efficient editing of the HBV genome by both Avec and Exci-Cas9

**Figure 2:**
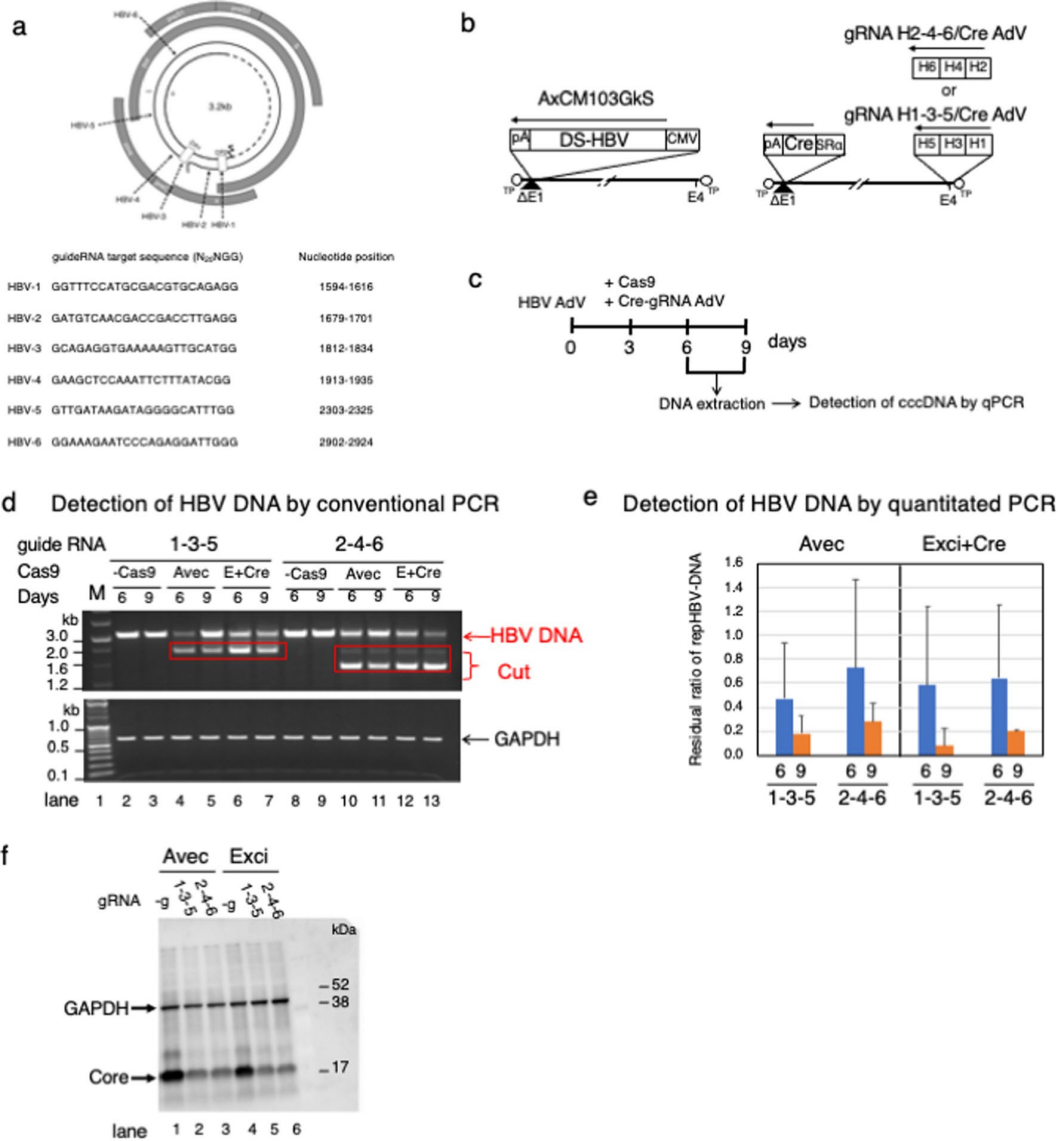
Efficient editing of the HBV genome by Exci-Cas9. (**a**) Position of guide RNA against the HBV genome. (**b**) AdVs for this experiment. Ax-CM103G-kS was used for the HBV103-AdV system. Two kinds of AdVs with three guide RNAs arranged in tandem were used. (**c**) Experimental schema. (**d**) Efficiency of genome editing as detected by conventional PCR using HBV primer sets (Supplementary Table S2) (The raw data was shown as Supplementary Fig. S5). HBV DNA indicates the remaining repHBV-DNA. Cut indicates DNA bands after repHBV-DNA genome editing. (**e**) Estimated remaining repHBV-DNA by TaqMan PCR using HBV primer–probe (Supplementary Table S2). Blue and orange columns show the values at 6 and 9 days after HBV-AdV transduction, respectively. The reproducibility was confirmed in two experiments, and the data of n = 3 was shown. (**f**) Remaining HBV Core protein after genome editing. GAPDH was used as an endogenous control.

In this study, as an example of genome editing by Exci-Cas9, we showed the efficiency of virus genome removal by targeting HBV, which is a DNA virus. Six guide RNAs for the HBV genome were designed (Fig. 2a). Two types AdVs carrying different gRNA sets and a Cre expression unit, namely gRNA H1-3-5/Cre AdV and gRNA H2-4-6/Cre AdV, were constructed (Fig. 2b, left). Detection of HBV genome replication was performed using a previously reported HBV103-AdV method^38^.

Ax-CM103G-kS (Fig. 2b, left), which carries an HBV genome modified to not express the S gene, was used to infect HepG2 cells. The AdVs expressing guide RNAs against the HBV genome and Cre were prepared as gRNA H1-3-5/Cre AdV having gRNA 1, 3, and 5 inserted in tandem, and gRNA H2-4-6/Cre AdV with gRNA 2, 4, and 6 (Fig. 2b, right).

Supplementary Fig. S3, because no band was detected 1 day after infection with Ax-CM103G-kS, shows that PCR using HBV primer sets (Supplementary Table S3) did not detect the HBV genome of the AdV, but only detected the HBV genomes generated after HBV replication. The replicated HBV genomes containing cccDNA (named repHBV-DNA) began to be detected on day 2 and were stably detected after 3 days. Therefore, Avec-Cas9 or Exci-Cas9 as Cas9 expression units and AdVs having gRNAs and Cre were used for coinfection at MOI 10 three days after Ax-CM103G-kS infection. Infected cells were harvested, and total DNAs were extracted at 6 and 9 days after Ax-CM103G-ks infection (Fig. 2c). A group infected with only gRNA-AdV was prepared as a control for genome editing (-Cas9).

The primer sets used for PCR reactions were shown in Supplementary Table S3. After successful genome editing, repHBV-DNA was excised between gRNAs. New bands derived from the shortened HBV genome resulting from genome editing were detected at about 2.0 kb using 1-3-5 gRNA and at about 1.5 kb using 2-4-6 gRNA (Fig. 2d). As a result, in both Avec (Fig. 2d, lane 4 for 1-3-5 and lane 10 for 2-4-6) and Exci (Fig. 2d, lane 6 for 1-3-5 and lane 12 for 2-4-6), shortened repHBV-DNA resulting from genome editing was detected from day 6, which was only 3 days after the introduction of Cas9. No difference depending on the gRNA was detected.

To quantify the amount of repHBV-DNA excised, the probe was set for the X gene coding region excised by Cas9, and quantitative PCR was performed. The residual amount of HBV-DNA after genome editing in each group is shown as a ratio when the Ct value of repHBV-DNA of the control, to which Cas9 was not added, was set as 1 (Fig. 2e). For Avec, the repHBV-DNA remaining on day 6 was 0.47 for g1-3-5 and 0.73 for g2-4-6, and on the ninth day it was 0.18 for g1-3-5 and 0.28 for g2-4-6. Similarly, for Exci-Cas9, the repHBV-DNA on day 6 was 0.58 for g1-3-5 and 0.64 for g2-4-6, and on the ninth day it was 0.08 for g1-3-5 and 0.20 for g2-4-6. This surprisingly showed that 80–90% of the HBV genome could be removed by not only Avec but also Exci-Cas9 at only 6 days after the introduction of Cas9. Between g1-3-5 and g2-4-6, g1-3-5 showed a slightly higher suppression rate, but no significant difference was observed.

Next, to show how much the expression of HBV protein was suppressed by genome editing using Avec or Exci-Cas9, HBV Core protein, which constitutes the virus particles, was detected by western blotting. We confirmed that both Avec and Exci-Cas9 efficiently suppressed the expression of Core protein (Fig. 2f).

### Application of the Exci-Cas9 system to cell-specific genome editing

The activity of cell-specific promoters is generally weaker than that of commonly used promoters. Previously, to overcome the weakness of cell-specific promoters, we reported a method using Cre. It consists of a combination of a switch unit that expresses Cre showing extremely high recombination efficiency from a cell-specific promoter, and a target unit constructed to express a gene of interest in a Cre-dependent manner. With this method, it was possible to increase the expression of a gene of interest by about 50 times while maintaining cell specificity as compared with using a cell-specific promoter directly^37^. Therefore, by applying Exci-Cas9, it may be possible to perform genome editing in a cell-specific manner.

**Figure 3:**
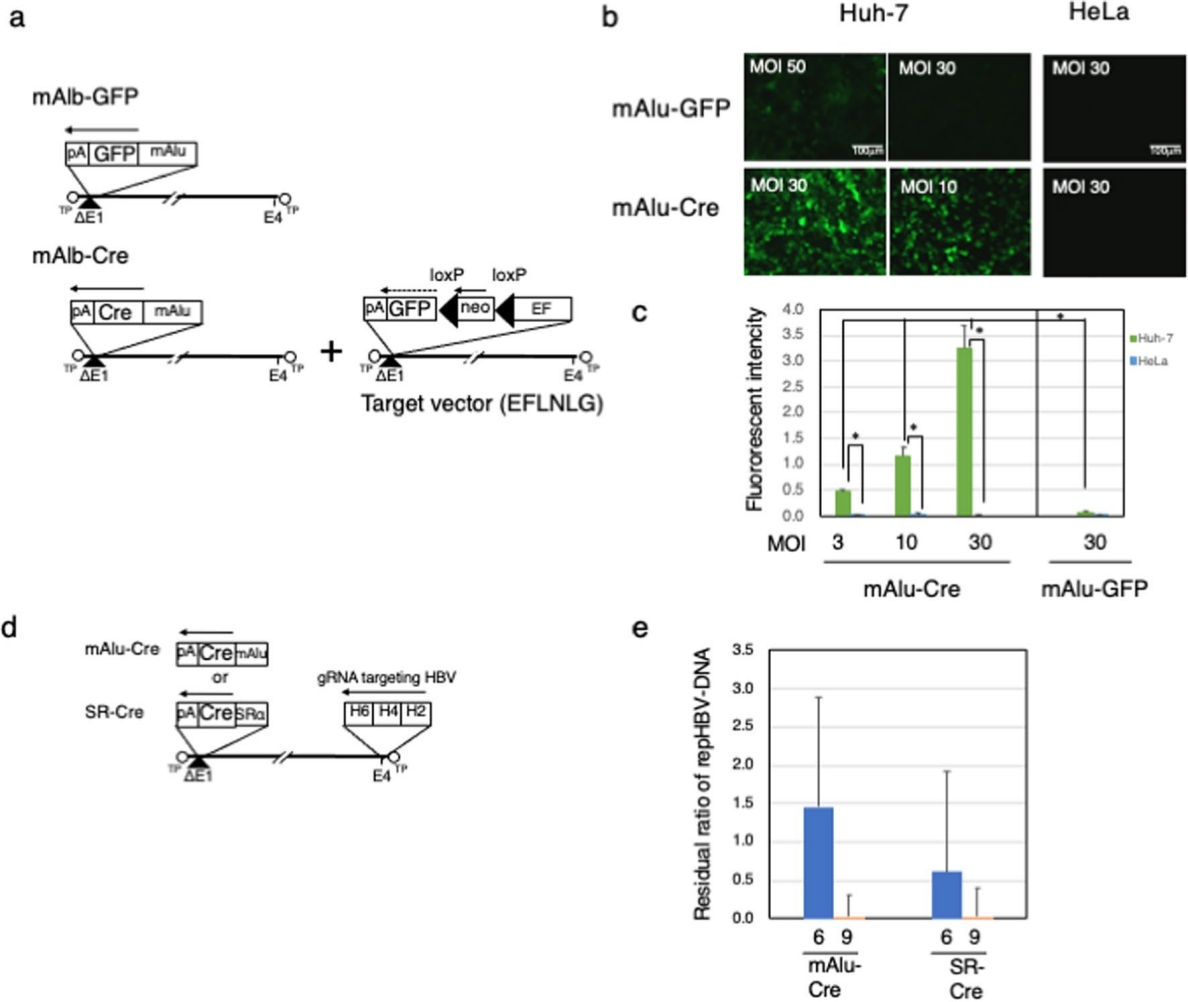
Cell-specific genome editing using Exci-Cas9. (**a**) AdVs to verify cell-specific promoter activity. (**b**) Fluorescence micrographs of Huh-7 or HeLa cells expressing GFP directly from the mAlu promoter or in a Cre-dependent manner expressed from the mAlu promoter. (**c**) Measured amount of GFP fluorescence. (post-test; *p < 0.0001). (**d**) AdVs for cell-specific genome editing of the HBV genome. mAlu-Cre or SR-Cre used the mAlu promoter or SRα promoter to express Cre, respectively. (**e**) Efficient elimination of HBV DNA by cell-specific Exci-Cas9. TaqMan PCR was performed in the same manner as in Fig. 2. The blue and orange columns show the values at 6 and 9 days after HBV-AdV transduction, respectively. The reproducibility was confirmed in two experiments, and the data of n = 3 was shown.

We chose the albumin promoter, which is hepatocyte specific, as a cell-specific promoter. mAlu-GFP, which has GFP expressed by the mouse albumin (mAlu) promoter, and mAlu-Cre, which has Cre expressed by the mAlu promoter, were constructed with AdV. As a Cre-dependent expression unit, we used a target AdV in which a stuffer region having a neomycin resistance gene between *lox*Ps inserted in the same direction was inserted between the EF1α promoter and GFP. Therefore, in this AdV, the expression of GFP depends on the expression of Cre (Fig. 3a).

In mAlu-GFP, the expression of GFP was barely seen at MOI 50. In contrast, in the target AdV with mAlu-Cre, sufficient GFP expression was seen even at MOI 10 (Fig. 3b). The GFP fluorescence intensity was quantified. As in a previous report, we found that the expression of GFP was more than 10 times higher in mAlu-Cre than in mAlu-GFP. In addition, regarding specificity, we clarified that the GFP intensity was below the detection limit in HeLa cells, which are not derived from hepatocytes (Fig. 3c).

Next, we prepared an AdV having mAlu-Cre and gRNA 2-4-6 against HBV (mAlu-Cre). The genome editing efficiency was compared between mAlu-Cre, which expresses Cre specifically in hepatocytes, and SR-Cre, which expresses Cre by the SRα promoter previously used in Exci-Cas9 (Fig. 3d)^39^.

In SR-Cre, the repHBV-DNA remaining on day 6 was 0.61, and on day 9 it was 0.13, which was almost the same as the results shown in Fig. 2. In mAlu-Cre, the repHBV-DNA on day 6 was 1.45, and no decrease of repHBV-DNA was detected. However, surprisingly, it was 0.10 on day 9, which was almost the same editing efficiency as SR-Cre (Fig. 3e). This difference between SR-Cre and mAlu-Cre was thought to be due to the difference in the amount of Cre expressed from the weak cell-specific promoter, especially on day 6. However, from day 9, mAlu-Cre showed almost the same HBV genome editing effect as SR-Cre in Huh-7 cells.

## Discussion

In this study, we reported an “Excisional-Cas9” system that showed strong Cas9 expression in a short period of time using AdV with high gene transduction efficiency in various cells. This system showed similar genome editing efficiency as Avec-Cas9, which continuously expresses Cas9 from AdV.

Genome editing has reached the clinical trial stage, but the possibility of off-target effects using a Cas9 stable expression system, for example AAV and lentiviral vectors, is a concern^2,40^. AdV is a transient expression vector, because it has been reported that adenovirus has less genome insertion into the chromosome^41^. However for the low-inflammatory vector that we previously reported^24^ or helper-dependent AdV^42^, the AdV genome is detected for 6 months extra-chromosomally. Therefore, even when using a transient AdV expression vector, the system in this study in which Cas9 is highly expressed for a short period is highly useful for genome editing. In the Exci-Cas9 system, AdV worked only as a tool to supply cells with plasmid-like circular double-stranded DNA. In other words, it was a system that can transfect target genes into 100% of cells and can be also applied in vivo. In fact, both the circular DNA with the Cas9 expression unit and the Cas9 protein derived from Exci-Cas9 disappeared in a shorter period compared to the AdV genome and Cas9 protein from Avec-Cas9 (Fig. 1). Although the expression period of Cas9 was shortened, the genome editing efficiency of GFP-expressing AdV was almost the same between Avec-Cas9 and Exci-Cas9 due to the use of a stronger promoter in the latter.

We chose the hepatitis B virus genome replication system to test Exci-Cas9. In HBV-infected cells, cccDNA, which is a template for viral transcripts and a cause of persistent infection, is stably present extra-chromosomally in the nucleus^28^. Since cccDNA is a viral genome, it has relatively little homology with the human genome. Thus, it may be safer to completely eliminate cccDNA from HBV-infected cells by genome editing than to target human chromosomes directly, and many attempts have been reported not only in vitro but also in vivo^43,44^. In in vitro analysis, Cas9 expressed by virus vectors shows significant cleavage of the HBV genome. And when the AAV vector has been used to express Cas9 in a liver-humanized mouse model, there has been highly significant improvement^29–35^. However, these reports acknowledge the need for more efficient genome editing methods. Exci-Cas9 may help to safely eliminate the HBV genome through editing^45^.

HBV genome replication is generally detected using HBV infection, cells with integrated HBV genomes, or plasmids having HBV genomes, but the replication efficiency of the HBV genome is low^46,47^. It has been reported that the HBV103-AdV system used in this study can induce highly efficient HBV genome replication because it introduce the HBV genome using AdV, which has high gene transfer efficiency, into hepatocytes^38^. Using this efficient HBV genome replication system, replicated HBV genomes were detected easily, which helped to perform experiments on genome editing.

But when examining the amount of repHBV-DNA after genome editing, it was possible that Cas9 cleaved the introduced HBV genomes by AdV and suppressed the supply of pre-genomic HBV RNA. In this study, Cas9 was introduced 3 days after HBV-AdV infection when repHBV-DNA was sufficiently detected (Supplementary Fig. S3). The efficiency of genome editing for HBV was as high as 80–90% compared to the efficiency for GFP, which was about 60%, so we considered that the decrease of pre-genomic HBV RNA was also a reason for the improvement of genome editing efficiency. However, we found that the genome editing efficiency against repHBV-DNA was also high.

Because the expression of Cas9 in Exci-Cas9 was short term, we made efforts to improve the efficiency of genome editing. First, we used AdV to increase gene transduction efficiency. As shown in Supplementary Fig. S1, the maximum indel induction obtained with plasmid transfection was achieved with an MOI of only 0.3 using AdV. Second was the use of the EF1α promoter, which shows high expression efficiency in mammalian cells, especially hepatocytes, to express Cas9 in Exci-Cas9^48,49^. Third, we used multiplex guide RNA. In fact, when quantifying indels using guide RNA for GFP, multiplex gRNA increased indel introduction efficiency about two-fold (Supplementary Fig. S2). Although adding multiple guide RNAs may increase the risk of off-target effects, we think that using multiplex guide RNA can increase the efficiency of genome editing. Fourth, SpCas9 is superior to SaCas9 not only for genome editing efficiency but also for freedom of guide RNA selection. SaCas9 requires a PAM sequence of six bases (NNGRRT), whereas that of SpCas9 is only three bases (NGG)^7,8^. AAV vectors typically use SaCas9 for genome editing due to size restrictions, but it is an advantage when AdV can use SpCas9.

One problem with Exci-Cas9 was that there was a time delay before genome editing began compared to Avec-Cas9. In the HBV replication system, Avec-Cas9 was more efficient for genome editing than Exci-Cas9 6 days after Cas9 transduction, although not significantly. In Exci-Cas9, recombination by expressed Cre is essential for generation of the Cas9 expression unit, but it was previously reported that it took about six hours to initiate recombination by Cre^50^. Because the results after 9 days showed that the cleavage efficiency of both were almost the same, this difference may be due to the time required for Cre expression. However, because the efficiency of eliminating multiple copies of repHBV-DNA in cells was almost the same between Exci-Cas9 and Avec-Cas9, this issue may be not important.

In addition, since Cre was used to induce Cas9 expression in Exci-Cas9, cell-specific genome editing was possible by expressing Cre driven by a cell- or tissue-specific promoter. There are several reports of Cas9 expressed by cell- or tissue-specific promoters, and clinical trials are also being conducted^2,51^. However, it is known that the activities of cell- or tissue-specific promoters are less than those of commonly used promoters^37^. As shown in Fig. 3, even with MOI 50, only slight GFP expression was detected in AdV, which expressed GFP directly from the albumin promoter. However, when AdV carrying Cre expressed from the albumin promoter and AdV having Cre-dependent expression of GFP from a strong promoter were combined, sufficient GFP expression was confirmed even with MOI 10, and cell specificity was strictly retained. We used only HeLa cells as a control to show specificity because it has already been reported by the study using replicative herpesvirus vector that the Alb promoter has liver specificity not only in vitro but also in vivo^52^.

We have already reported that the expression level of a target gene using the α-fetoprotein promoter was increased by about 50 times using this system. Considering that the Cas9 expression level is important for the success of genome editing and that it is possible to reduce the amount of vector transduced to obtain sufficient Cas9 expression, this method may be useful for cell- or tissue-specific genome editing.

In conclusion, the Exci-Cas9 system is useful not only for strong expression of Cas9 for a short period, but also to enable highly efficient cell- or tissue-specific genome editing. We believe that Exci-Cas9 may be useful not only to reduce off-target effects due to constitutive Cas9 expression, but also to eliminate persistent DNA viruses in addition to HBV.

## Materials and methods

### Cell culture, transfection, and AdV infection

Human cell lines 293, HeLa, Huh-7, and HepG2 are derived from embryonic kidney, cervical carcinoma, hepatocellular carcinoma, and hepatoblastoma, respectively. 293 and HeLa cells were cultured in Dulbecco’s Modified Eagle Medium (DMEM) supplemented with 10% fetal calf serum (FCS). 293 cells constitutively express adenoviral E1 genes and support the replication of E1-substituted AdVs. Huh-7 and HepG2 cells were kept in high glucose DMEM supplemented with 10% FCS.

Lipofectamine^R^ LTX and PLUS™ Reagent was used for plasmid transfection. The transfection was performed according to the manufacturer’s protocol, and medium was exchanged after 24 h.

After infection with AdVs, cells were maintained in DMEM supplemented with 5% FCS.

### Plasmid and AdV construction for CRISPR/Cas9

All Cas9 gene expression unit was derived from pX330 (Addgene plasmid #44230), which encodes hSpCas9 and gRNA. Cas9 under the control of the CBh promoter was excised as a *Kpn*I–*Not*I fragment and inserted into the *Swa*I cloning site at the E1 substitution region of the cosmid cassette pAxdV-FVF-4c containing the full-length AdV genome, resulting in Avec-Cas9^53–55^.

Exci-Cas9 possesses an identical structure to AxALNLZCAL, except that LacZ gene is replaced by a Cas9 gene excised as an *Age*I-*Eco*RI fragment^37^. Briefly, Cas9 and rabbit β-globin poly(A) are located ‘upstream’ of the CAG promoter between two *lox*P sequences, and the expression unit was inserted into the *Swa*I cloning site at the E1 substitution region of the cosmid cassette pAxdV-FVF-4c.

Three gRNA sequences targeting the GFP gene and six gRNA sequences targeting the HBV genome were selected using online design software at http://crispr.mit.edu to decrease the risk of undesirable off-target mutations in the host genome^1^. The gRNA sequences are described in Supplementary Table S1 for GFP and in Fig. 2a for the HBV genome, respectively. All gRNAs were expressed by the human U6 promoter. Complementary oligonucleotides were annealed and ligated into the *Bbs*I site of pX330 and designated Cas9/gRNA G-1 plasmid, Cas9/gRNA G-2 plasmid, and Cas9/gRNA G-3 plasmid.

The Cre recombinase gene tagged with a nuclear localization signal under the control of the SRα promoter was inserted into the *Swa*I cloning site at the E1 substitution region of the cosmid cassette pAxdV-FVF-4c containing the full-length AdV genome, resulting in pAxSRNCre-4c^50^. Fragments of the gRNA expression unit were inserted into the E4 cloning site of pAxSRNCre-4c located in the E4 region 162 nucleotides downstream from the right end of the adenovirus-5 (Ad5) genome, and the resulting cosmids were designated gRNA G-1/Cre AdV, gRNA G-2/Cre AdV, and gRNA G-3/Cre AdV. The fragments of multiplex gRNA expression cassettes were constructed using polymerase chain reaction (PCR) and restriction enzymes that produce non-palindromic termini, and were introduced into the E4 cloning site of pAxSRNCre-4c^56^. All plasmids were confirmed by DNA sequencing.

All plasmids and adenovirus vectors can be provided by Y. Kanegae.

### AdV purification and titration

GFP-AdV possesses a GFP expression unit under the control of the EF1α promoter at the E1 substitution region^57^.

The AdV called Ax-CM103G-kS has the HBV genome of genotype C (accession number AB246345) and is designed to increase the amount of replicated HBV genome in cells by having a mutated ATG of Small S. Ax-CM103G-dP carries a defective HBV genome lacking part of the polymerase domain essential for HBV genome replication. These HBV AdVs were generated as described in Suzuki et al.^38^. Purified and concentrated viral stocks were prepared as described in Kanegae et al.^58^.

AdVs were titrated using the method described by Pei et al.^59^. Briefly, copy numbers of viral genomes that were successfully transduced into the infected target cells were measured using real-time PCR (relative virus titer: rVT). Huh-7 cells or HepG2 cells were used as the target cells. The titer of the standard virus was determined using the copy number of serially diluted plasmid DNA. The rVT (copies/ml) normally corresponds to about one-fifth of the 50% tissue culture infectious dose (TCID_50_) in a titration or plaque assay. The sequences of the TaqMan probes for the titration were derived from Ad5 pIX gene (Supplementary Table S2).

### Conventional PCR

PCR experiments were essentially performed by standard methods^60^. Briefly, HepG2 cells in 24-well plates were infected with AdVs and incubated for 6- and 9-days post-infection. Total DNAs were extracted and amplified by PCR with the Tks Gflex DNA polymerase and PCR system. The PCR cycling conditions were in accordance with the manufacturer’s protocol: 94 °C for 1 min, followed by 26 cycles at 98 °C for 10 s, 60 °C for 15 s, and 68 °C for 30 s, and a final extension at 72 °C for 4 min. The primer sets are described in Supplementary Table S3.

### T7 endonuclease 1 (T7E1) assay

Huh-7 cells were harvested 6-days post-infection. Total DNAs were extracted and amplified by PCR as described above, except using 30 PCR cycles. The sequences of the primers are described in Supplementary Table S3. Heteroduplex DNA was formed by heating and cooling PCR products in a thermal cycler and incubating with T7E1 according to the manufacturer’s instructions. Products were resolved by 2% agarose gel, and DNA band intensities were acquired with ImageJ software. Indels (%) were calculated as previously reported^61^.

### Quantitative real-time PCR

To quantify the remaining DNA amounts of the Cas9 gene or replicated HBV genome per cell, TaqMan PCR was applied. Briefly, after infection by Cas9-expressing AdV, cells were cultured for the designated number of days, harvested, and total DNA was extracted using MagExtractor Genome. For an endogenous control, the amount of chromosomal DNA was simultaneously measured by human β-actin primer/probe (Supplementary Table S2). The qPCR reaction was performed according to the manufacturer’s protocol: 50 °C for 2 min and 95 °C for 10 min, followed by 40 cycles of 95 °C for 15 s and 60 °C for 1 min using QuantStudio™ 5^37,38^.

### Western blotting

Each day after infection, HepG2 cells were harvested, and the total protein was extracted using NP-40 lysis buffer [50 mM Tris–HCl (pH 8.0), 0.15 M NaCl, 5 mM EDTA, 1% NP-40]. The lysates were mixed well in a rotator for 2 h at 4 °C, centrifuged at 15,000 rpm for 5 min at 4 °C, and supernatants were collected. Western blotting was performed as described previously^62^. Membranes were incubated for 2 h at room temperature in the presence of anti-Core rabbit antibody (kindly gifted from Dr. Suzuki, Hamamatsu University School of Medicine) diluted to 1/100 or anti-Flag mouse monoclonal antibody (Merck KGaA., #4042 Darmstadt, Germany) diluted to 1/2000 with PBS-Tween. For an endogenous control, anti-GAPDH rabbit monoclonal antibody (Cell Signaling Technology, #2118, MA, USA) diluted 1/1000 were also used. After washing, membranes were incubated with peroxidase-conjugated anti-rabbit IgG (H + L) (Promega Co., #W401B, WI, USA) diluted 1/1000 or peroxidase-conjugated anti-mouse IgG (H + L) (Promega Co., #W402B) diluted 1/5000 with PBS-Tween, respectively.

## Supplementary Information

Supplementary Information.
