# Supplementary material for: Short term but highly efficient Cas9 expression mediated by excisional system using adenovirus vector and Cre
Source: Sci Rep. 2021 Dec 21;11:24369. doi: 10.1038/s41598-021-03803-w (PMC8692473; doi:10.1038/s41598-021-03803-w)

Supplemental table S1

Guide-RNA sequence targeting GFP

	guideRNA target sequence (N_{20}NGG)	Nucleotide position from ATG
G1	GGGCGAGGAGCTGTTCACCGGGG	12-34
G2	GCCGTCCAGCTCGACCAGGATGG	41-63
G3	GGCCACAAGTTCAGCGTGTCCGG	73-95

Supplemental table S2

Primers for quantitative PCR (TaqMan PCR)

Target	Orientation	Sequence (5'-3')
ZCas9	Forward	AGCGACATCCTGAGAGTGAACA
	Reverse	TGCCGCACGAGAGCTTTC
	[FAM]probe	CTGAGCGCCTCTATGA
AdV	Forward	TGTGATGGGCTCCAGCATT
	Reverse	TCGTAGGTCAAGGTAGTAGAGTTTGC
	[FAM]probe	ATGGTCGCCCCGTCCTGCC
GFP	Forward	GCGATTTCGATTTCCGGTCGCC
	Reverse	GGCACGGGCAGCTTGCCGGTGGTGC
	[FAM]probe	GTGCCCATCCTGGTCGAGCTG
HBV	Forward	CGGCACCGACAACTCTGTT
	Reverse	GCTGTATGGTGAGGAGAACAATGTT
	[FAM]probe	CACTTCGCTTCACCTCTGCAC
ß-actin	Forward	CTCGCAGCTCACCATGGAT
	Reverse	ATGCCGGAGCCGTTGTC
	[VIC]probe	ATGATATCGCCGCGCTCGTCGT
GAPDH	Human GAPD Endogenous Control (VIC™/TAMRA™ probe, primer limited) (4310884E) (Thermo Fisher Scientific Inc.)	

Supplemental table S3

Primers for conventional PCR

Target	Orientation	Sequence (5’-3’)
GFP	Forward	TCTCGAGCTTTTGGAGTACGTCGTCTTTAG
	Reverse	GGCACGGGCAGCTTGCCGGTGGTGC
HBV	Forward	GCGCACCTCTCTTTACGCGGTCTCC
	Reverse	GACGTCCCGCGCAGGATCCAGTTGGCAGCAC
GAPDH	Forward	CGCGCTTAGCACCCCTGGCCAAGGTCATCC
	Reverse	GCGTCTAGACGGCAGGTCAGGTCCACCAC

Supplementary Figure S1

Comparison of genome editing efficiencies between AdV and plasmid transfection

(a) Structure of AdV and plasmid used in the experiment. (b) Indel induction by AdV or plasmid. Uncut indicates bands derived from the target DNA without genome editing. Cut indicates bands that appeared after genome editing. Bands were quantitated by Image J, and indels (%) were calculated as described in the Materials and Methods.

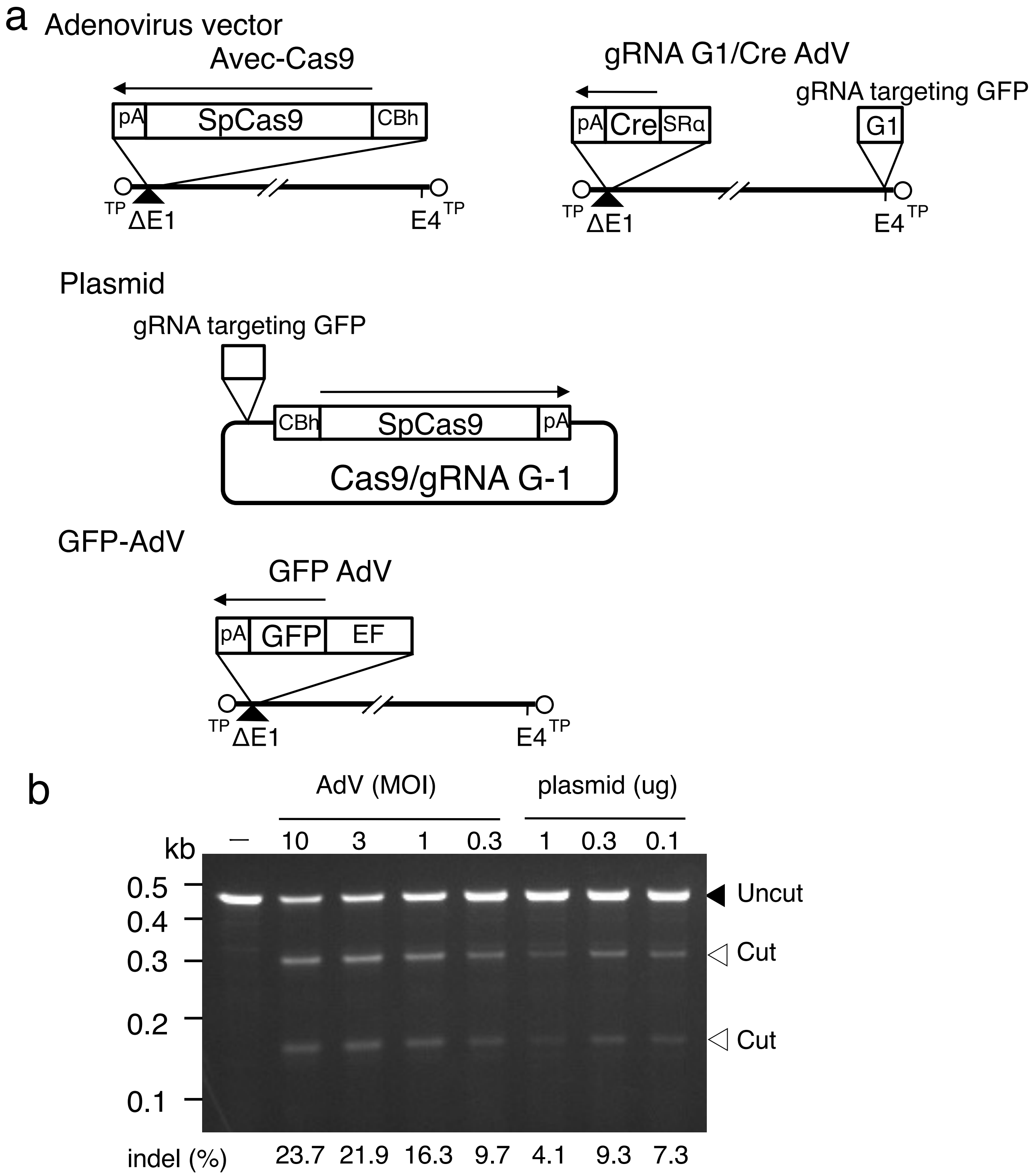

Supplementary Figure S2

Multiplex guide RNA prior to single guide RNA

One days after GFP-expressing AdV infection, gRNA-G2/Cre AdV, gRNA-G3/Cre AdV or gRNA-G2-3/Cre AdV and Avec-Cas9 were infected in Huh-7 cells. Five days after, Cells were harvested, total DNA extracted and analyzed by T7E1 assay. (post-test; *p < 0.0001)

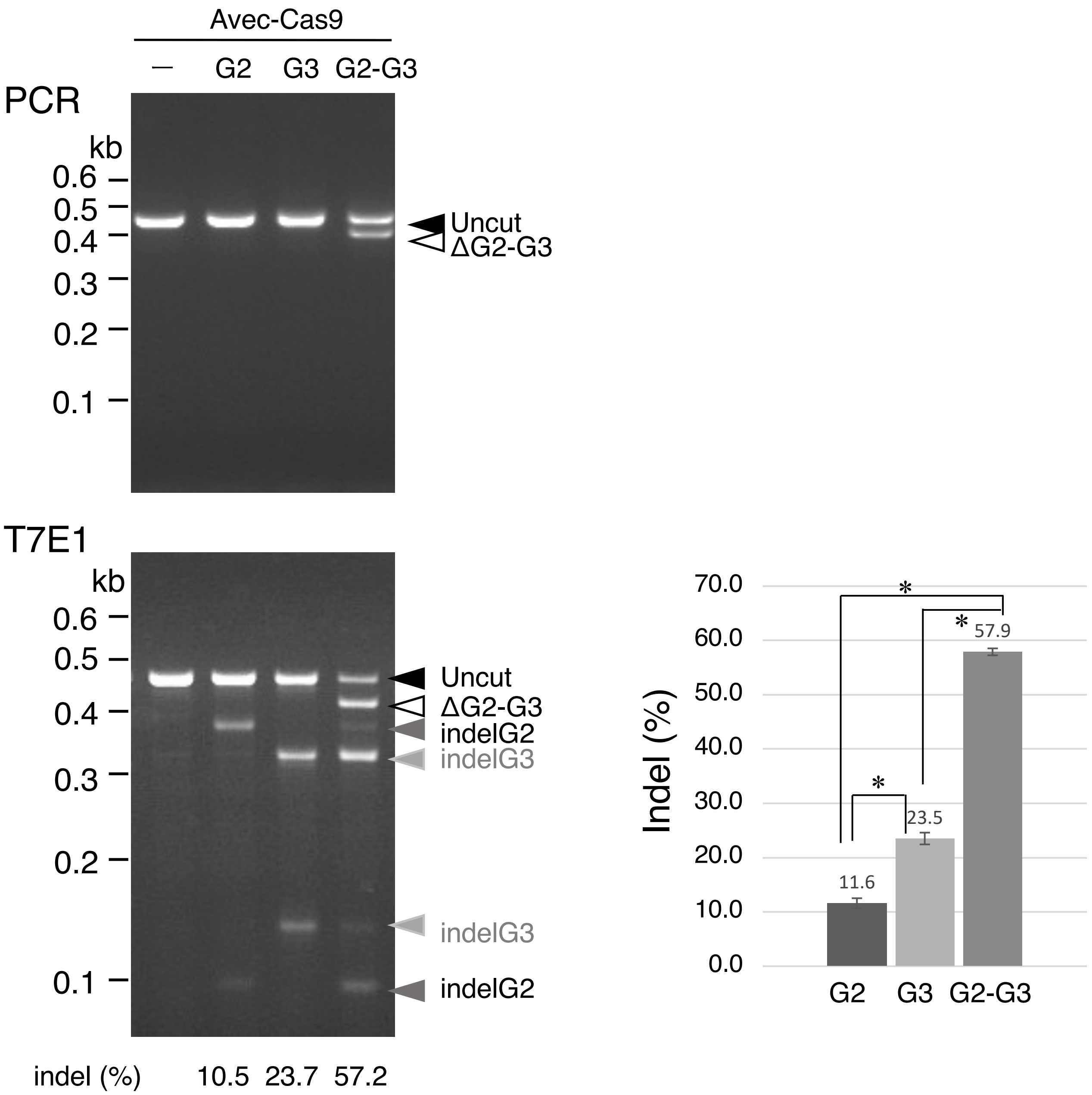

Supplementary Figure S3

Confirmation of repHBV-DNA by HBV103-AdV system

Cell: HepG2

Primer: HBV (Supplementary File S2)

beta-actin F:CACCATTGGCAATGAGCGGTTC

R:AGGTCTTTGCGGATGTCCACGT

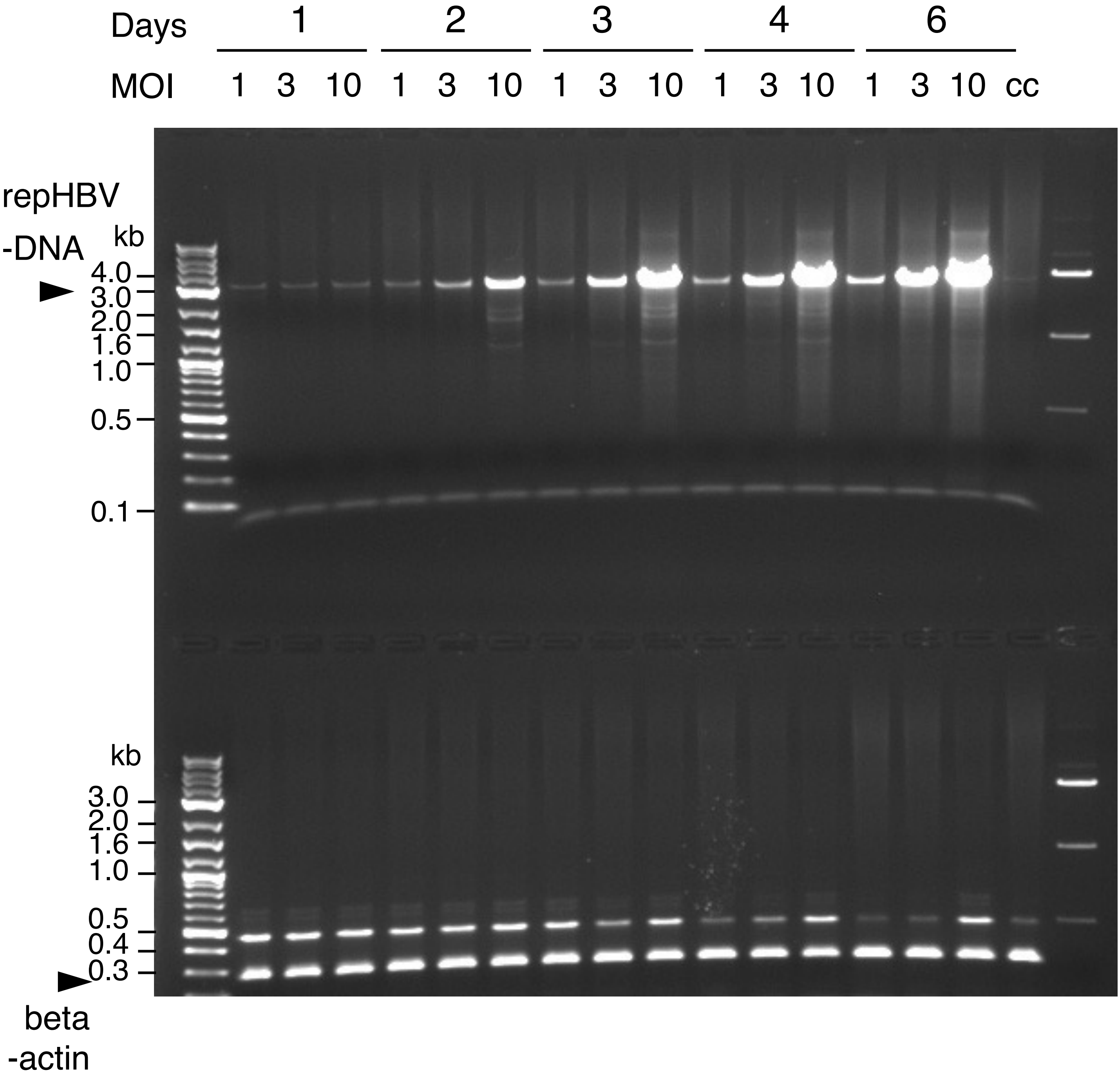

Supplementary Figure S4

Time course of expressed Cas9 protein.
GAPDH was used as an endogenous control.
Figure 1d full-length western blots.

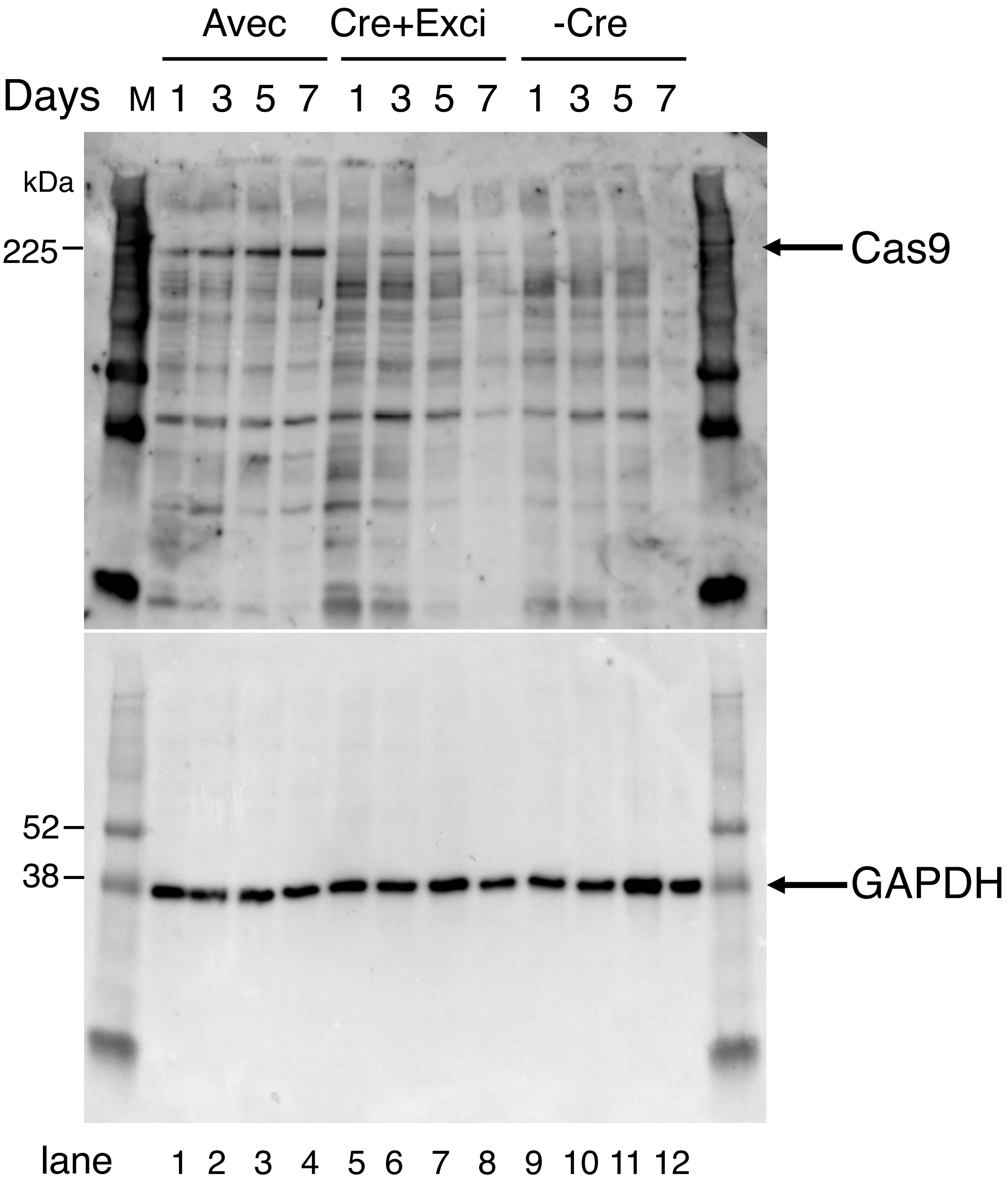

Supplementary Figure S5

Detection of HBV DNA by conventional PCR

Cell: HepG2

Primer: HBV, GAPDH (Supplementary File S2)

Raw data in Figure 2d

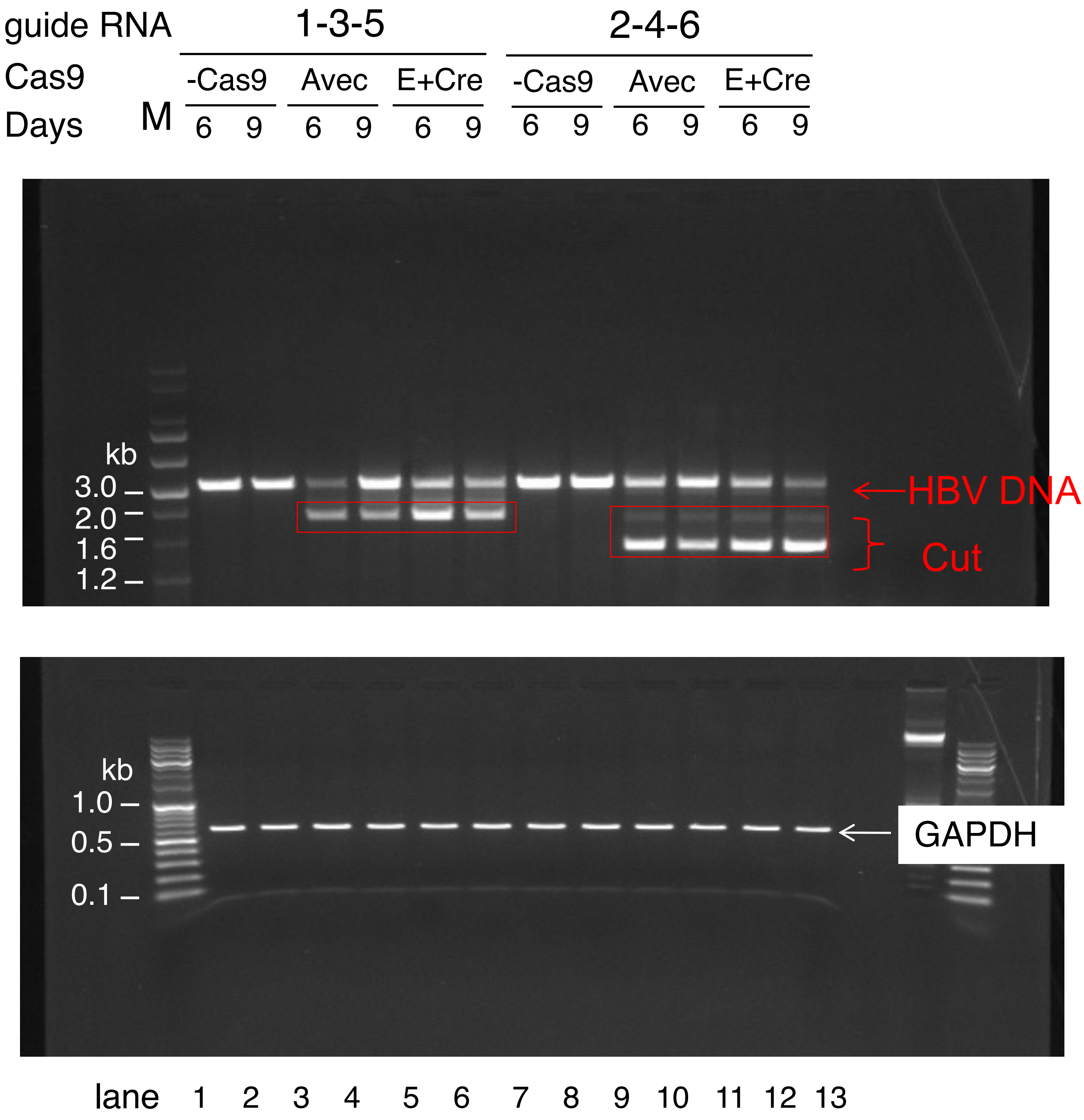

Supplement: Supplementary file 1 — Supplementary Information. [file 41598_2021_3803_MOESM1_ESM.pdf]
